# Traumatic cardiac arrest in the emergency department– Overview upon primary causes

**Published:** 2014-06-25

**Authors:** V Georgescu, O Tudorache, V Strambu

**Affiliations:** * “Carol Davila" Clinical Nephrology Hospital, Bucharest; ** “Sf. Pantelimon" Emergency Hospital, Bucharest; *** “Carol Davila" University of Medicine and Pharmacy, Bucharest, “Carol Davila" Clinical Nephrology Hospital, Bucharest

**Keywords:** Cardiac arrest, blunt trauma, ISS, thoracic trauma

## Abstract

Abstract

Rationale: Trauma is the leading cause of death for patients aged less than 40 years. Trauma patients with cardiac arrest have low survival rates, the resuscitation being often considered futile and consumptive of medical and human resources.

Objective: The aim of this study is to describe the main characteristics in cases of patients critically traumatized, who were admitted in our emergency department.

Methods and Results: The study is based on a retrospective analysis of cases of major trauma admitted in an Emergency Department between 2004 and 2008. There were 201 cases of critically traumatized patients, who received cardiopulmonary resuscitation. The patients were aged between 16 and 79, mostly men (67.16%), with a range of ISS between 30 and 75. Regarding the type of mechanism that produced the injury we noted a predominance of blunt trauma (87,2% of cases) and hypovolemia as a direct cause that led to the cardiac arrest. The first monitored rhythm was non-shockable for over 90% of the cases. In our group, 4 patients were discharged alive (2% of all cardiac arrest cases). The mechanism of cardiac arrest for those 4 cases were hypoxia through massive facial trauma in one case and tension pneumothorax through severe thoracic trauma in three cases.

Discussion: Given the low survival figures, all the efforts that could be achieved by an emergency team in the face of severe trauma had to be oriented towards the maintaining of the vital functions or, when needed, towards restoring life in order to enrich the operation theatre for the definitive care.

## Introduction

Trauma is the leading cause of death for patients aged below 40 years [**1**]. Chest injuries, injuries of the heart and of the great vessels are responsible for about 25% of the post-traumatic deaths and count as an aggravating factor for another 25% of the trauma-related deaths [**2**]. Patients with cardiac arrest caused by any condition may have survival rates up to 17% after cardiopulmonary resuscitation [**3**], whilst trauma patients with cardiac arrest, as most studies show, survive only in rates of 0% to 3.7% [**4**]. Therefore, resuscitation in trauma patients is considered by many respectable researchers to be futile and consumptive of medical and human resources, especially in the field and in case of multiple victims [**5**]. National Association of EMS Physicians and American College of Surgeons Committee on Trauma have produced guidelines that sustain withholding resuscitation in out-of-hospital setting of posttraumatic cardiac arrest [**4**]. Early recognition and treatment of causes leading to cardiorespiratory arrest may improve the patient’s outcome and prevent cardiac arrest. 

 The aim of this study is to describe the main characteristic causes in cases of patients critically traumatized who were admitted in our emergency department. Based on the survival rates, a predominance of a certain cause of cardiac arrest was established, characteristic of survival patients, we had to concentrate the resuscitative efforts so that to delay the occurrence of cardiac arrest and to treat the cause.

## Methods

The study is based upon a retrospective analysis of cases of major trauma admitted in the emergency department of “Sf. Pantelimon" Emergency Hospital in Bucharest between 2004 and 2008. The patients admitted in the study were adults (aged over 16), critically traumatized, who suffered from cardiorespiratory arrest in the emergency department. All the patients received cardiopulmonary resuscitation according to resuscitation protocols that were in use at that moment. The patients who died at the moment of arrival (dead on scene, or during transportation) were not included in the study. 

 The data collected for this study are those mentioned in the Utstein style charts completed for all the cases of cardiorespiratory arrest, specifically designed for our emergency department and in the ordinary, official charts of the emergency department, selected for critically traumatic patients. The Injury Severity Score (ISS) was calculated for every patient.

## Results

There were 201 cases of critically traumatized patients complicated with cardiopulmonary arrest, who received cardiopulmonary resuscitation (CPR) in the emergency department between 2004 and 2008. Aged between 16 and 79 with a median age of 41, we found that more men, 135 cases (67.16%), than women – 76 (32.84%) were treated in our department. The range of ISS was between 30 and 75, which emphasized the severity of the trauma.

 The emergency department team performed all the necessary maneuvers for the evaluation and treatment of all trauma patients regarding oxygenation, establishing definitive and secure airway, intravenous access and administration of fluids, performing Advanced Life Support. 

 Bilateral chest decompression was performed in 20 cases and there were no thoracotomies for great vessels compression or internal cardiac massage in the emergency department. 15 patients were surgically treated in the operating room after the restoration of spontaneous circulation (ROSC), through CPR.

**Fig. 1:**
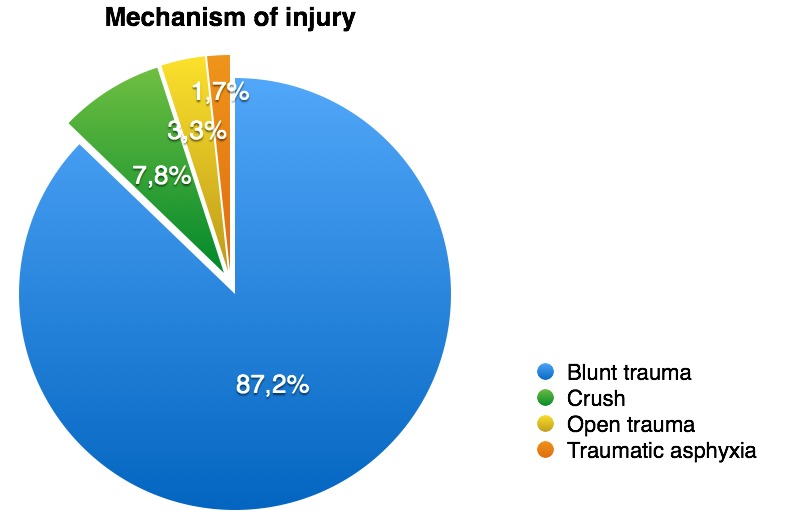
Mechanism of injury

 Regarding the type of mechanism which produced the injury we noted a real predominance of blunt trauma – there were 87,2% of cases, the rest of them consisting of crush, open trauma or traumatic asphyxia (**Fig. 1**).

**Fig. 2:**
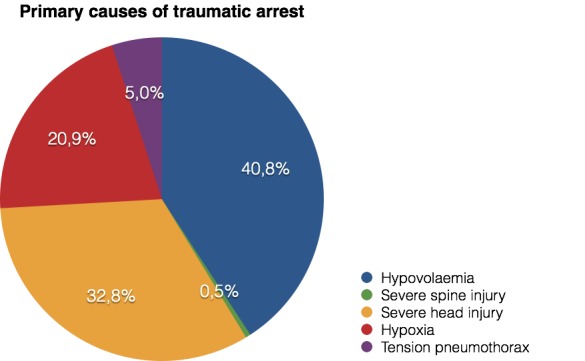
Primary causes of traumatic arrest

 When talking about the direct cause that conducted to the cardiac arrest, it can be said that there is a predominance of hypovolemia, with 40,8% of cases, followed by severe head injury, and (**Fig. 2**).

**Fig. 3:**
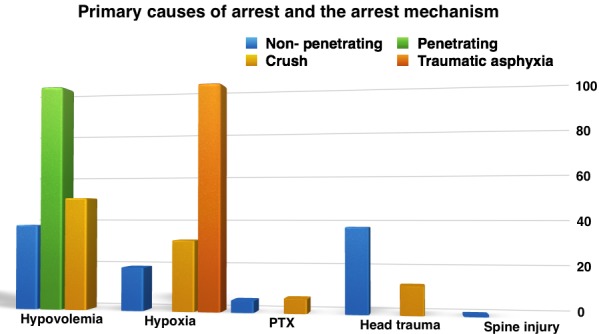
Causes of arrest correlated with mechanism of injury

 The primary causes of arrest correlated with the mechanism of injury for our study are depicted in Fig. 3.

**Fig. 4:**
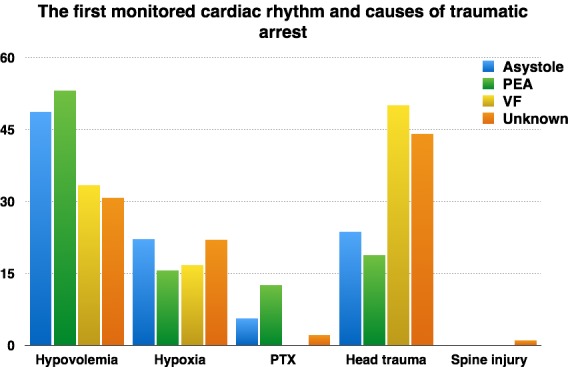
First monitored rhythm

 It is known that the rhythm through which the heart stops influences the further evolution of cardiorespiratory arrest and, even in our study, we revealed the correlation between the first monitored rhythm and the mechanism of collapse. Hypovolemia, for instance, is characterized by the dominance of pulseless electrical activity (PEA) and asystole in 53,1% and 48,6% of cases, respectively, while ventricular fibrillation dominates the mechanisms of cardiac arrest in cases of severe head injury, with 50% of the cases, as it can be noticed in Fig. 4.

**Fig. 5:**
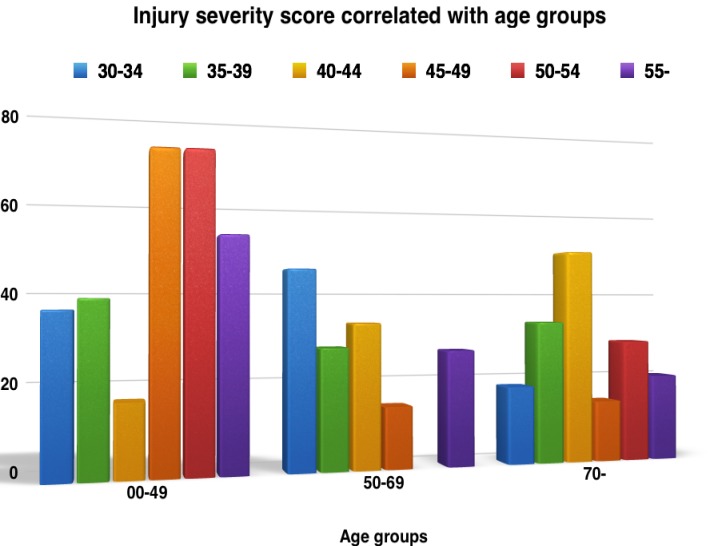
ISS correlated with age groups

 Considering the value of ISS when we appreciated the prognosis of a certain trauma patient, despite the capacity of ISS in predicting this with precision, we correlated the calculated values of ISS at the moment of admission in our study with age groups, for instance (**Fig. 5**)

 As it is depicted in Fig. 5, the age group 45-49 and 50-54 appeared to be the most representative (71,4%) for trauma characterized by a less severe ISS– 25-36. We did not encounter the same values in the group aged 40-45 years; the ones who appeared to be the age group with the highest risk in terms of trauma, were the ones with ISS values around 70.

 One of most used terms used to measure the success of a resuscitation effort, traumatic or non traumatic, is the Restoration (Return) of Spontaneous Circulation (ROSC), which means that the event due to which the heart beat again, with the function of pump restored, revealed by the presence of carotid pulse after a resuscitative intervention. If it is prolonged for over 20 minutes, the event will be considered as “Survived event".

**Table 1:** ROSC achieved in 180 cases

ROSC	YES	NO	TOTAL
<20’	0	149	149
Row %	0	100	100
Col %	0	84,7	82,8
>20'	4	27	31
Row %	12,9	87,1	100
Col %	100	15,3	17,2
TOTAL	4	176	180
Row %	2,2	97,8	100
Col %	100	100	100

**Fig. 6:**
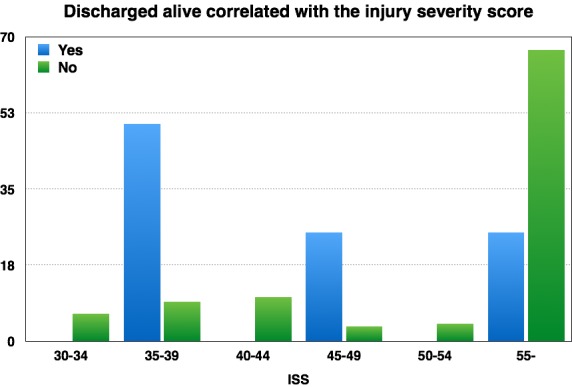
Patients discharged alive and injury severity score

 In our study, ROSC was achieved in 180 cases and in 31 cases it was more than 20 min. Very important, although modest in numeric values, was the successful resuscitation itself, with 4 survivors or discharged alive, which means 2% of all cardiac arrest cases (**Table 1**). The mechanism of cardiac arrest for those 4 cases which were successfully resuscitated were hypoxia through massive facial trauma in one case and tension pneumothorax through severe thoracic trauma in three cases. On this occasion, we studied the correlation between ISS and discharged alive and we found that most patients who left the hospital alive are from that young group (aged 35-39) despite a high ISS. In the same manner, as it could be predicted, that a raised ISS is more dangerous for an old patient and those who were discharged alive and had a minor ISS. This is revealed in Fig. 6. 

## Discussions

The resuscitation of the patients who suffered from trauma-related cardiac arrest remains a controversial topic. Some studies showed higher rates of survival (7.5%), in the context of an urban setting, physician-led system [**6**], while other studies considered trauma-related cardiac arrest resuscitation futile [**7**]. Our study showed a 2% survival rate for the cases included. 

 The population represented by the cases included in the study showed that men are more frequently exposed to this kind of events, the median age found was 41, and asystole was the first monitored rhythm for most of the patients. These characteristics are similar to the ones described in larger studies [**8**]. 

 It can also be said that ISS measured severity of trauma correlated with the most active ages, socially speaking, as, in our study the younger patients were given a higher ISS score. 

 The insertion of the chest tube for the tension pneumothorax proved to be survival factor, such as 3 of the alive discharged patients in our study benefited from the maneuver. This situation is mentioned as a strong prediction factor in the survival in traumatic cardiac arrests [**9**]. 

 The different correlations between certain variables in order to assess the probability of the presence of a certain cause in face of a certain condition are interesting things to observe. Therefore, it can be revealed that hypovolemia characterizes the main mechanism of cardiac arrest in case of penetrated traumatisms and blunt trauma and crush trauma as well, being the leading cause of collapse among the all causes analyzed. This observation is confirmed by studies and guidelines, as well [**10**]. 

 Given the low survival figures, all efforts that could be achieved by an emergency team in the face of severe trauma, no matter the place, the appreciated risk of survival, in terms of age, of gravity of lesions (ISS or other scores), the supplies or quality of training, must be oriented to maintaining the vital signs, the vital functions or, when needed, to restoring life in order to enrich the operation theatre for final treatment, definitive care.
